# Accelerometer-assessed physical behavior and the association with clinical outcomes in implantable cardioverter-defibrillator recipients: A systematic review

**DOI:** 10.1016/j.cvdhj.2021.11.006

**Published:** 2021-11-24

**Authors:** Maarten Z.H. Kolk, Diana M. Frodi, Tariq O. Andersen, Joss Langford, Soeren Z. Diederichsen, Jesper H. Svendsen, Hanno L. Tan, Reinoud E. Knops, Fleur V.Y. Tjong

**Affiliations:** ∗ Heart Center, Department of Clinical and Experimental Cardiology, Amsterdam UMC, Academic Medical Center, Amsterdam, the Netherlands; † Department of Cardiology, Copenhagen University Hospital – Rigshospitalet, Copenhagen, Denmark; ‡ Department of Computer Science, University of Copenhagen, Copenhagen, Denmark; § Activinsights, Cambridgeshire, United Kingdom; ‖ Vital Beats, Copenhagen, Denmark; ¶ Department of Clinical Medicine, Faculty of Health and Medical Sciences, University of Copenhagen, Copenhagen, Denmark; # Netherlands Heart Institute, Utrecht, the Netherlands; ∗∗ College of Life and Environmental Sciences, University of Exeter, Exeter, United Kingdom

**Keywords:** Accelerometry, Implantable cardioverter-defibrillator, Physical behavior, Systematic review, Ventricular tachyarrhythmia

## Abstract

**Background:**

Current implantable cardioverter-defibrillator (ICD) devices are equipped with a device-embedded accelerometer capable of capturing physical activity (PA). In contrast, wearable accelerometer-based methods enable the measurement of physical behavior (PB) that encompasses not only PA but also sleep behavior, sedentary time, and rest-activity patterns.

**Objective:**

This systematic review evaluates accelerometer-based methods used in patients carrying an ICD or at high risk of sudden cardiac death.

**Methods:**

Papers were identified via the OVID MEDLINE and OVID EMBASE databases. PB could be assessed using a wearable accelerometer or an embedded accelerometer in the ICD.

**Results:**

A total of 52 papers were deemed appropriate for this review. Out of these studies, 30 examined device-embedded accelerometry (189,811 patients), 19 examined wearable accelerometry (1601 patients), and 3 validated wearable accelerometry against device-embedded accelerometry (106 patients). The main findings were that a low level of PA after implantation of the ICD and a decline in PA were both associated with an increased risk of mortality, heart failure hospitalization, and appropriate ICD shock. Second, PA was affected by cardiac factors (eg, onset of atrial fibrillation, ICD shocks) and noncardiac factors (eg, seasonal differences, societal factors).

**Conclusion:**

This review demonstrated the potential of accelerometer-measured PA as a marker of clinical deterioration and ventricular arrhythmias. Notwithstanding that the evidence of PB assessed using wearable accelerometry was limited, there seems to be potential for accelerometers to improve early warning systems and facilitate preventative and proactive strategies.

**Key Findings**

• Implantable cardioverter-defibrillator (ICD) devices are equipped with a device-embedded accelerometer capable of capturing physical activity (PA), whereas wearable accelerometer–based methods enable the measurement of physical behavior that encompasses activity, sleep behavior, sedentary time, and rest-activity patterns.
• In this systematic review, 52 studies that evaluated accelerometer-based methods in patients carrying an ICD or at high risk of sudden cardiac death were summarized.
• The main findings were that a low level of PA after implantation and a decline in PA were both associated with an increased risk of mortality, heart failure hospitalization, and appropriate ICD shock.
• This systematic review demonstrates the potential of accelerometer-measured activity as a marker of clinical deterioration; however, future prospective research and long-term collection of wearable accelerometry data are required to gain better understanding of the predictive value of physical behavior.

## Introduction

Patients at a high risk of life-threatening ventricular arrhythmias and sudden cardiac death (SCD) benefit from preventative implantable cardioverter-defibrillator (ICD) implantation.^1^ However, ICD carriers are at risk of frequent hospital readmission after implantation, increased mortality rates, and psychological distress due to appropriate and inappropriate ICD shocks.^2^, ^3^, ^4^, ^5^, ^6^, ^7^, ^8^ The role of physical behavior (PB) is investigated in an attempt to find new predictors and markers of clinical deterioration. Contrary to physical activity (PA), which focuses only on body movement that requires energy expenditure, PB is an umbrella term for an individual’s behavior and activities throughout day and night, which also includes sleep, daily activities, posture, rest-activity patterns, and sedentary behavior.^9^^,^^10^ Accelerometers enable the continuous and objective quantification of daily PB by the recording of body movement along reference axes and signal analysis (ie, intensity, frequency, and velocity of activity and postural changes).^11^^,^^12^ There is growing interest in accelerometry-based methods such as wearable accelerometry and accelerometers embedded in ICD devices, the latter capable of capturing PA only. If specific patterns in PB were to be identified and related to the likelihood of clinical outcomes, this could improve early warning systems and enable physicians to take proactive and preventative measures to avert clinical deterioration. Nevertheless, no comprehensive overview of literature regarding the full spectrum of PB in ICD carriers has yet been undertaken.^13^ A systematic review was therefore conducted to evaluate published literature on accelerometer-measured PB in an ICD or high-risk SCD population. We addressed the following question: What is the clinical value of PB for identification of clinical deterioration leading to ICD therapy, heart failure (HF) hospitalization, and mortality?

## Methods

This review was reported according to the Preferred Reporting Items for Systematic Reviews and Meta-Analyses (PRISMA) statement, as outlined in the protocol beforehand.^14^

### Literature search

The MEDLINE® (Ovid) and EMBASE® (Ovid) electronic databases were systematically searched to identify studies published between January 2000 and August 2020. Both databases were searched on September 1, 2020 using the terms “implantable cardioverter defibrillator,” “sudden cardiac death,” “heart failure,” and “accelerometer.” The full search strategy is provided in the supplemental material (Supplemental Tables 1 and 2). The search strategy, including terms and limits, was designed in collaboration with a medical information specialist. The reference lists of relevant papers were hand-searched to identify studies potentially missed by the electronic search.

### Eligibility criteria

The following inclusion and exclusion criteria were applied to each identified record to determine eligibility. First, patients who received an ICD with or without cardiac resynchronization therapy (CRT-D) or a wearable cardioverter-defibrillator (WCD) for primary or secondary prevention of SCD were included. Second, patients at high risk of SCD but who had not undergone ICD or CRT-D implantation were included, in essence patients diagnosed with HF (New York Heart Association [NYHA] class II–IV and left ventricular ejection fraction [LVEF] ≤35%), a primary [inherited] arrhythmia syndrome [eg, long QT syndrome], or a cardiomyopathy [eg, hypertrophic cardiomyopathy, dilated cardiomyopathy, arrhythmogenic right ventricular cardiomyopathy]). Accelerometer-based methods considered eligible were either wearable (body-worn) accelerometry or device-embedded accelerometry. Outcomes of interest included ICD therapy for ventricular arrhythmias (defibrillator shock or antitachycardia pacing), HF hospitalization, mortality, functional status (eg, NYHA class) and quality of life. Studies were excluded if these were not performed under free-living conditions or in subjects <18 years old. Animal studies, case reports, small case series (n < 10), conference abstracts, and secondary studies were excluded. Only articles that were published in peer-reviewed journals in the English language and where full text was available were included. Titles and abstracts of identified records, and the full text of potentially relevant records, were evaluated by 2 independent reviewers (M.K. and D.F.) in a blinded fashion. Any disagreement between the 2 reviewers was resolved through discussion; a third reviewer (F.T.) was consulted when no consensus was reached.

### Data collection and extraction

A data charting form was jointly developed by 2 reviewers (M.K. and D.F.) to determine which variables to extract. Collaboratively the reviewers tabulated the data, discussed the results, and updated the data iteratively. Predefined characteristics were extracted from included studies: authors, publication year, study period, study design, number of participants, accelerometer specifications (vendor and version, number of axis, wear site), wear time, primary endpoints, and follow-up duration. Means of the study participants’ age and percentage of male patients were calculated. Studies were grouped by type of accelerometer used (ie, device-embedded or wearable accelerometer). Effect sizes and 95% confidence intervals (CI) for study endpoints ICD therapy, HF hospitalization, mortality, and composite endpoints were extracted. Hazard ratios were inverted to be interpreted as hazard of events with decreasing levels of physical activity.

### Quality assessment

The Quality in Prognosis Studies (QUIPS) tool was used for the appraisal of methodological quality of the included studies.^15^ The tool consists of 6 domains: study participation, study attrition, prognostic factor measurement, outcome measurement, study confounding, and statistical analysis and reporting. These domains were scored on their risk of bias (low, moderate, high, unknown). A summated risk of bias was determined for each study according to the suggestions from Hayden and colleagues^15^ and Lazzerini and colleagues.^16^ The quality assessment was performed by 2 independent reviewers (M.K. and D.F.). Any initial disagreement was settled through discussion, and if needed by consulting the third reviewer (F.T.).

## Results

**Figure 1:**
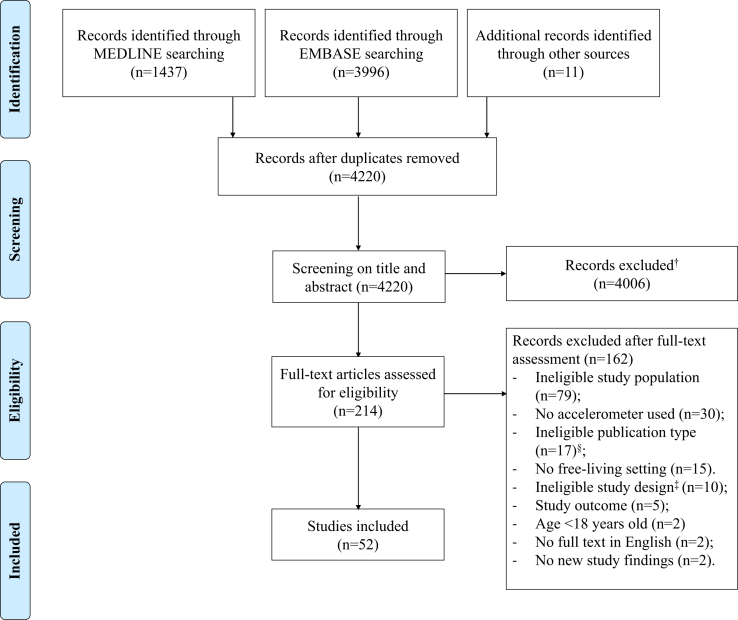
Study selection flow chart showing the results in each step of the systematic search to identify studies. ^†^Exclusion of abstract-only and conference abstracts (n = 148). ^‡^Case study, case series. ^§^Editorial, review, study protocol, or rationale.

The MEDLINE (n = 1437) and EMBASE (n = 3996) database searches returned a total of 4209 unique articles after removal of duplicates (n = 1224). Another 11 articles were identified through scrutiny of reference lists of relevant studies. After screening of title and abstract, 214 articles were identified for full-text screening. Most frequently reported reasons for exclusion were: ineligible study population (n = 79), no accelerometer used (n = 30), ineligible publication type or study design (n = 27) and a non-free-living study setting (n = 15). Figure 1 displays a flow diagram of the study selection process. Ultimately, a total of 52 studies were included in this review. Out of these studies, 30 studies examined device-embedded accelerometry (189,811 patients), 19 studies examined wearable accelerometry (1601 patients), and 3 studies validated wearable accelerometry against device-embedded accelerometry (106 patients). The study population consisted of ICD and/or CRT-D patients (35 studies), WCD carriers (3 studies), and high-risk SCD patients (14 studies). The device-embedded accelerometer measured only physical activity (D-PA), whereas wearable accelerometry studies reported on different dimensions of PA (eg, energy expenditure, time spent in different PA intensities, peak performance, and measures of variability), sedentary time, sleep behavior, and rest-activity patterns. The wearable accelerometers most commonly used were ActiGraph GT3X+ (7 studies), Actiwatch-64 (3 studies), and Actiwatch 2 (2 studies). The most common wear site was the wrist (10 studies), followed by waist (8 studies) and ankle (1 study). Wear times generally ranged between 3 days and 14 days. An overview of all included studies is provided in the supplemental material (Supplemental Tables 3–5). The risk-of-bias assessment for each study evaluated is displayed in Supplemental Figure 1 and Supplemental Table 6. In total, 22 studies were scored as low risk of bias, 19 as moderate risk, and 8 studies as high risk. Studies by Melczer and colleagues,^17^ Pressler and colleagues,^18^ and Shoemaker and colleagues^19^ reported only on the validity of accelerometry and were excluded from the quality assessment.

### Device-measured physical activity

**Figure 2:**
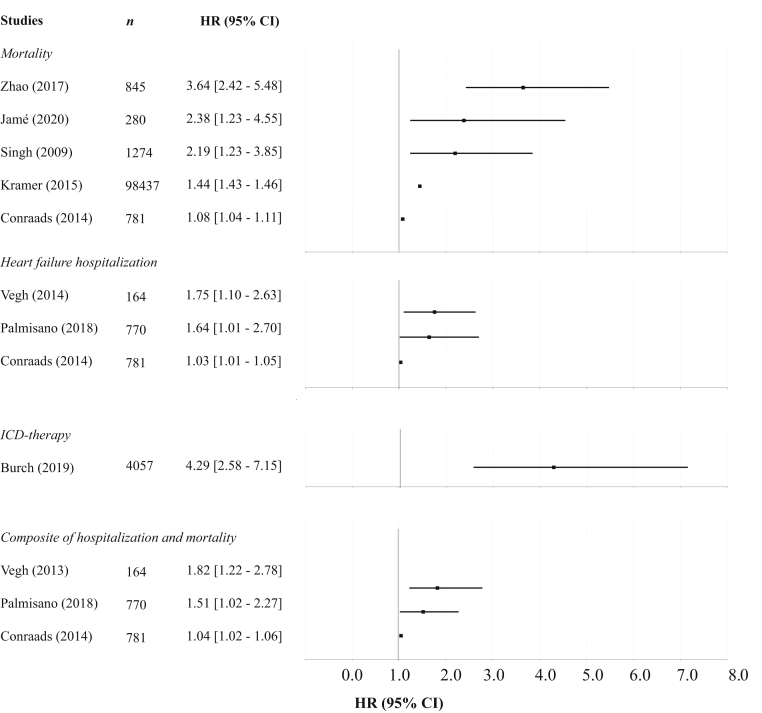
Association between low physical activity after implantation and mortality, heart failure (HF) hospitalization, implantable cardioverter-defibrillator (ICD) therapy, and composite endpoints. HR = hazard ratio. Note: Vegh et al included cardiac transplant and left ventricular assist device in the composite endpoint.

In total, 30 studies examined device-embedded accelerometry (cumulative 189,811 patients, mean age 69 years, 72% male). A summary of observations and related clinical outcomes is provided in Table 1. In general, an increase in D-PA following implantation of an ICD, after initiation of CRT, and during wear time of the WCD was observed; this increase reached a plateau approximately 12 weeks postimplantation.^20^, ^21^, ^22^, ^23^ Seven studies illustrated that patients in whom D-PA remained low following device implantation (in a range between 3 days and 2 months following implantation) were at an increased risk of mortality, HF hospitalization, and the composite endpoint of HF hospitalization and mortality during follow-up (Figure 2).^24^, ^25^, ^26^, ^27^, ^28^, ^29^, ^30^ Similarly, a retrospective study demonstrated that low D-PA during the first week of WCD use alone was associated with an increased risk of appropriate defibrillator shock.^31^ Furthermore, 10 studies found an increased risk of mortality, HF hospitalization, or a composite of mortality and hospitalization in patients with declining levels of D-PA over time (ranging from 8 weeks to 4 days prior to event) compared to patients with stable activity levels.^24^^,^^25^^,^^32^, ^33^, ^34^, ^35^, ^36^, ^37^, ^38^, ^39^ Of these studies, 5 tested a prediction algorithm for hospitalization and/or mortality within 30 days, where D-PA was among the included variables with sensitivities ranging from 34% to 90.5%.^34^, ^35^, ^36^, ^37^^,^^39^ In a study by Perego and colleagues,^40^ low D-PA during 1-year follow-up was associated with an increased risk of HF hospitalization. In terms of defibrillator therapy, in a retrospective cohort consisting of female patients, D-PA started to decline 16 days prior to the ventricular arrhythmia and defibrillator shock.^23^ On the contrary, in an observational study by Sears and colleagues^22^ D-PA levels in patients who experienced an ICD shock during follow-up did not differ significantly from patients who did not receive ICD therapy. Last, 5 studies described factors that affected D-PA levels.^22^^,^^41^, ^42^, ^43^, ^44^ Two observational studies illustrated seasonal differences in D-PA and a decline in D-PA during the COVID-19 pandemic lockdown.^42^^,^^44^ Further, a significant reduction in D-PA was observed following defibrillator shock and the onset of persistent atrial fibrillation.^22^^,^^41^^,^^43^

**Table 1:** Summary of observations and related health effects in studies that examined device-embedded accelerometry

Observation	Effect	N	Study design (no. studies)	Follow-up	References
Low PA following device implantation					
	Increased risk of mortality	101,617	RCT substudy (1);	12–31 months	24, 25, 26, 27, 28
			Registry (2);		
			Retrospective observational (1);		
			Prospective observational (1)		
	Increased risk of hospitalization	1715	RCT substudy (1);	15–36 months	28, 29, 30
			Registry (1);		
			Prospective observational (1)		
	Increased risk of atrial arrhythmias	770	Retrospective registry (1)	25 months	29
	Increased risk of ICD shock	4057	Retrospective observational (1)	1 month	31
	Increased risk of combined endpoint HF hospitalization or mortality†	1715	RCT substudy (1);	15–36 months	28, 29, 20
			Registry (1);		
			Prospective observational (1)		
Decline in PA					
	Increased risk of mortality	126,234	RCT substudy (1);	26–28 months	24,25,32,33
			Registry (2);		
			Retrospective observational (1)		
	Increased risk of hospitalization	3522	Prospective observational (5)	11.7–17 months	34, 35, 36, 37,40
	Increased risk of shock‡	4927	Retrospective observational (1)	1 month	23
	Increased risk of combined endpoint HF—hospitalization or mortality	22,312	RCT substudy (1);	12–60 months	32,38,39
			Registry (1);		
			Prospective observational (1)		
Noncardiac factors					
	Season variation affects PA	102	Retrospective observational (1)	12 months	44
	Pandemic lockdown reduces PA	24	Retrospective observational (1)	80 days	42
Cardiac factors					
	ICD therapy reduces PA	2944	Data from RCT (1);	12–22 months	22,43
			Prospective clinical trial (1)		
	Atrial fibrillation onset reduces PA	266	Prospective observational (1)	51.6 months	41

HF = heart failure; ICD = implantable cardioverter-defibrillator; PA = physical activity; RCT = randomized controlled trial.

† Vegh et al included transplant and left ventricular assist device in the composite endpoint.

‡ Sears et al found no difference in physical activity only.

### Wearable accelerometry

A total of 19 studies (cumulative 1601 patients, mean age 65 years, 69% male) that evaluated wearable accelerometry were included. A summary of observations and related clinical outcomes is provided in Table 2. These studies predominantly reported metrics related to PA and sedentary behavior (13 studies), followed by sleep behavior (5 studies) and rest-activity patterns (1 study). First, Liebzeit and colleagues^45^ found that patients with HF had significantly dampened rest-activity patterns: HF patients demonstrated a lower mean activity and activity range and a flat circadian rhythmicity compared to healthy adults, which suggests that patients do not reach the high levels of daytime activity observed in healthy adults. A similar finding was observed in patients with ischemic HF, who spent 67% of their time in low-intensity activities, while the time spent in vigorous-intensity activities was low (4.7%).^46^ Compared to HF patients, ICD carriers walked more steps per day and reached a higher peak performance (a daily average of the highest step rate values).^47^ Apart from the study by Witham and colleagues,^48^ there was a significant difference in daily total step count and time spent in moderate-to-vigorous physical activity (MVPA) across NYHA class groups^49^, ^50^, ^51^ and a correlation between step count and LVEF was seen.^52^ Five studies demonstrated that sleep behavior and PA were associated with patient-reported physical function, quality of life, and cognitive function.^49^^,^^52^, ^53^, ^54^, ^55^

**Table 2:** Summary of observations and related health effects in studies that examined wearable accelerometry

Observation	Effect	N	Study design (no. studies)	References
Low physical activity	Increased risk of hospitalization and mortality	286	RCT substudy (2); Prospective observational (1).	56, 57, 58
HF patients engage in low-intensity activity, have a poor objective sleep continuity and flat circadian rhythmicity	N/A	174	Cross-sectional (3);	45,46,74
Step count and MVPA associated with NYHA class and LVEF†	N/A	456	RCT substudy (1); Cross-sectional (2); Pilot study (1); Feasibility study (1)	48, 49, 50, 51, 52
High step count, MVPA, and sleep time associated with patient-reported QoL, functional status, and cognitive function	N/A	331	RCT substudy (3); Feasibility study (1); Cross-sectional (1)	49,52, 53, 54, 55

HF = heart failure; ICD = implantable cardioverter-defibrillator; LVEF = left ventricular ejection fraction; MVPA = moderate–to-vigorous physical activity; N/A = not applicable; NYHA = New York Heart Association; PA = physical activity; QoL = quality of life; RCT = randomized controlled trial.

† Witham et al did not show an association between NYHA class and accelerometry counts.

Furthermore, 3 studies evaluated the relationship between accelerometer-derived variables and clinical endpoints. First, Prescher and colleagues^56^ tested the prognostic value of an accelerometer-based quantification of a 6-minute walking test in a home environment for patients with advanced HF. Patients with a low number of steps and a short distance during the walking test at baseline were at higher risk of HF hospitalization or mortality during a mean follow-up of 15 ±6 months.^56^ Second, Evangelista and colleagues^57^ evaluated the incidence of hospitalization and mortality in HF patients participating in a 6-month, home-based exercise program. Participants who improved in the distance walked during follow-up were at a reduced risk of the composite endpoint of all-cause hospitalization and mortality during 12 months follow-up.^57^ Third, Melin and colleagues^58^ examined the effect of adding measures of variability related to activity (peak skewness and kurtosis) to the HF survival score. The addition of peak skewness to the model significantly improved the predictive ability during a median follow-up duration of 3 years.

### Validation studies comparing wearable and device-embedded accelerometry

In total, 3 studies investigated the validity of device-embedded accelerometry compared to wearable accelerometry. Although there were moderate-to-strong intrapersonal correlations between wearable accelerometry (triaxial accelerometer) and device-embedded accelerometry (uniaxial accelerometer), studies reported large variations in total daily activity between device-embedded accelerometry and wearable accelerometry (Supplemental Table 5).^17^, ^18^, ^19^ There was an underestimation of approximately 0.8 hours in daily activity measured using device-embedded accelerometry compared to validated wearable accelerometers.^18^^,^^19^

## Discussion

This systematic review displays the body of evidence with respect to the utility of accelerometer-measured PB in ICD carriers and high-risk SCD patients. First, the findings illustrate that a low PA following device implantation and a decline in PA (ie, time-varying activity) are associated with an increased risk of ICD therapy, HF hospitalization, mortality and the composite endpoint of mortality and hospitalization. Second, the level of PA is affected by noncardiac factors (eg, seasonal variation and societal factors) and cardiac factors (eg, ICD shocks and onset of atrial fibrillation). This review adds a comprehensive overview of the role of device-embedded and wearable accelerometry in patients at high risk of SCD with or without an ICD, and has included several large observational studies published over the last years.^21^^,^^23^^,^^25^^,^^31^^,^^38^ In a systematic review by Rosman and colleagues^13^ where only the role of device-embedded accelerometers was investigated, similar associations between D-PA and clinical outcomes were found. A systematic review by Tan and colleagues^59^ demonstrated that PA measured using wearable accelerometry has independent predictive value for mortality and hospitalization in HF patients. This is the first systematic review that has examined the full spectrum of PB by aggregating results from both wearable accelerometry and device-embedded accelerometry.

### Physical activity and early warning systems

Patient selection and risk stratification for ICD implantation is currently based on left ventricular functionality, NYHA class, and life expectancy.^1^ However, these markers are insufficient to avoid ICD implantation in patients who are at greater risk of nonarrhythmic death after implantation and device complications (inappropriate shock, infection, and more) than of ventricular arrhythmias and appropriate ICD therapy.^60^^,^^61^ Furthermore, hospitalization and early mortality remain common in the ICD population despite technical improvements and implementation of telemonitoring.^3^^,^^4^^,^^62^ This review has demonstrated that low PA following device implantation and a decline in PA are both associated with ICD therapy, HF hospitalization, and mortality. Hence, PA as a surrogate for functional capacity could reflect worsening of functional status and be a marker of an increased risk of clinical events and mortality. Therefore, accelerometer-assessed PA could serve as a marker of clinical deterioration, especially in an ICD population. On the other hand, it has also become apparent that other factors—both cardiac-related and noncardiac—affect the level of PA, apart from clinical worsening alone. Despite several large studies indicating strong relationships between PA and clinical outcomes, these studies did not consistently account for covariates such as age, sex, cardiovascular medication, comorbidities, and HF severity (NYHA class, LVEF). Also, these inferences were based on observational data and fail to prove causation. For instance, it has been hypothesized that the decline in PA after ICD shocks could be a result of a complex relationship between psychological and biological factors that can lead to altered behavior.^22^ Moreover, environmental changes have been shown to be a precipitating factor for HF decompensation and ventricular arrhythmias, but it remains uncertain whether changes in PB due to noncardiac factors lead to increased risk of clinical deterioration.^63^, ^64^, ^65^ Future prospective studies are needed to simultaneously address the effect of cardiac and noncardiac factors on PB and clinical endpoints in order to gain insight in causality.

### Future directions

Based on this systematic review, we propose 3 directions for future research. First, a steep increase in the amount of data collected in ICD carriers has been observed over the past years, derived from data sources such as device remote monitoring systems, consumer- and research-level activity trackers, and electronic health records. However, the clinical utility of these data remains unclear. By leveraging machine learning (ML) techniques, the enormous amount of personalized time series data could be used for accurate prediction models and precision medicine. Marzec and colleagues^66^ designed a prediction model for ventricular tachycardia episodes based on D-PA data, albeit application of various ML techniques did not render any added predictive value compared to random chance. An ML prediction model by Shakibfar and colleagues^67^ based on data from remote monitoring of the ICD showed that decline in D-PA levels 4 days prior to the onset of electrical storm was among the most relevant features and yielded an area under the curve of 0.80. Hence, the integration of accelerometer-assessed metrics among other features may lead to accurate real-time prediction of impending cardiac events at a high accuracy.

Second, this review indicates that the number of studies that have examined device-embedded accelerometry currently outweighs that of wearable accelerometry in an ICD population; nevertheless, there are important limitations to device-embedded accelerometry. In addition to the initial use of device-embedded accelerometers for rate-responsiveness pacing, ICD manufactures have used their proprietary algorithms to collect daily summaries of PA. Out of 30 studies evaluating device-embedded accelerometry, 13 reported on the threshold for discrimination between activity and inactivity based on an acceleration exceeding a preset fixed threshold (equivalent to approximately 3.2 km/h or a step rate of 70–80 steps per minute). Aside from the underreporting of the applied thresholds and problematic generalizability of these data, a recent study by Dibben and colleagues^68^ demonstrated that HF-specific accelerometer intensity thresholds for (in)activity were substantially lower (< 50%) than commonly used. Raw accelerometer data obtained using wearable accelerometers, however, are universal and can be converted into specific metrics such as the performance during the most active period of the day, measures of variability, rest-activity patterns, sleep behavior, time spent in different intensities of activity, and sedentary time. Also, raw accelerometer data can be translated to specific activities such as daily activity (vacuuming, cleaning windows), sedentary positions (lying, sitting), stair climbing, cycling, and running with the use of ML techniques.^69^ In Table 3, the multitude of metrics obtained using wearable accelerometers in this review in comparison to device-embedded accelerometers is displayed. The emerging paradigm of *time-use epidemiology* revolves around the interactions of behavior and the integration of a variety of continuously collected metrics, meaning that all behaviors are necessarily related to each other and should not be collected in isolation.^9^ In the absence of device-embedded accelerometers capable of collecting raw accelerometer data, specific cut-off values and intensity thresholds have to be derived from calibration studies and related to adverse cardiac outcomes to avoid underestimation and misclassification of PA and enhance generalizability of the findings. Moreover, this review has demonstrated substantial variations in total daily activity measured using uniaxial device-embedded accelerometry and validated triaxial wearable accelerometry. Triaxial accelerometers capture acceleration in 3 directions that may lead to more precise measurement of activity, compared to device-embedded accelerometers that use a single-axis accelerometer.

**Table 3:** Domains and metrics captured by wearable and device-embedded accelerometers

Type of accelerometer	Domain	Metric
Wearable accelerometry	Physical activity	Energy expenditure, steps per day, time spent at different intensities of activity, peak performance, cadence, measure of variability (skewness, kurtosis)
	Sleep behavior	Total sleep time, percentage wake after sleep onset, sleep onset latency, nocturnal activity, sleep efficiency
	Sedentary behavior	Sedentary time
	Rest-activity pattern	Amplitude (range of activity, difference between maximum and minimum level), mesor (24-hour mean activity), acrophase (time of peak activity during 24 hours), and R-squared value in cosinor analysis (circadian rhythmicity)
	Posture	Standing, sitting, and lying position
	Adherence	Time wearable is worn per day
		
Device-embedded accelerometry	Physical activity	*Implantable cardioverter-defibrillator*:
		Time active per day
		*Wearable cardioverter-defibrillator*:
		Step count, body position

Third, considering the association of high or increased PA with improved clinical outcomes, one could question if a causal relation exists or if PA merely serves as a marker for physical fitness and good clinical status. Three meta-analyses showed a significantly lower likelihood of ICD shock and better cardiorespiratory fitness in patients participating in exercise programs,^70^, ^71^, ^72^ suggesting that high or increased PA could be a potential modifiable risk factor. Future research is needed to elucidate the direct effect of increased PA on clinical outcomes and the impact of PA as a modifiable risk factor, potentially providing new preventative strategies for signaling a decline in health status and offering timely medical interventions to reduce the patient’s risk.

### Limitations

To our knowledge this is the first comprehensive review evaluating accelerometry-based methods in a high-risk SCD population, with or without an ICD. A broad overview of the current state of evidence was displayed by including a wide range of study designs and different methodologies. The risk of bias was reduced using 2 independent investigators for study selection and data charting, an exhaustive search, and a study protocol designed a priori. However, there are several limitations to acknowledge. First, the lenient eligibility criteria have resulted in heterogeneity among study designs, study populations, and endpoints. Subsequently, primary arrhythmia syndromes were only marginally represented compared to studies focused on patients with diagnosed HF. Also, there was an imbalance in sample size between studies (range between 10 and 98,437 patients) and the number of studies that evaluated device-embedded accelerometry and wearable accelerometry (respectively, 30 and 19 studies). Second, the majority of included studies were observational or substudies from experimental study designs. Although several studies have accounted for possible confounders, our quality assessment showed study confounding to be a frequent risk of bias (Supplemental Figure 1). Last, in general studies examined wearable accelerometry up to 14 days, albeit long-term longitudinal accelerometry data are critical to capture cyclical trends and overall patterns in variability and to fully understand the relationships between different behavioral patterns and the effect of reallocation of specific behavior on clinical outcomes.^73^

## Conclusion

The adoption of commercial and research-grade accelerometers has resulted in an abundance of continuously collected data. This study provides an overview of the wide range of studies using accelerometer-based methods in patients at high risk of SCD and ICD carriers, and proposes future directions for research. In conclusion, there may be value of accelerometry as a tool for improving follow-up care for ICD patients; however, the mechanisms behind and potential causal relations between low and decreasing PA and clinical deterioration warrant further research. Future prospective research and long-term collection of wearable accelerometry data are required to gain better understanding of the clinical utility and predictive value of PB in an ICD or high-risk SCD population.

## Funding Sources

We thank the European Union funding program for research and innovation for the Horizon 2020 (grant number: Eurostars project E!113994- SafeHeart).

## Disclosures

The authors (Tan, Knops, Andersen, Svendsen, Tjong, and Langford) have received funding for the SafeHeart study through the EUROSTARS PROJECT E!113994 Horizon 2020 grant. The funding source has no involvement in study design; collection, analysis, and interpretation of data; or writing or publishing of the study. Andersen is co-founder of Vital Beats and has stock ownership. Andersen is co-author of a pending patent application that is within the field of this study. Langford is an employee and shareholder of Activinsights Ltd, the manufacturers of the behavioral assessment wearable used in the study. FT received consulting honoraria from Boston Scientific and Abbott. Authors Frodi, Kolk, and Diederichsen have no conflicts to disclose.

## Authorship

All authors attest they meet the current ICMJE criteria for authorship.
