# Supplementary material for: Accelerometer-assessed physical behavior and the association with clinical outcomes in implantable cardioverter-defibrillator recipients: A systematic review
Source: Cardiovasc Digit Health J. 2021 Nov 24;3(1):46–55. doi: 10.1016/j.cvdhj.2021.11.006 (PMC8890329; doi:10.1016/j.cvdhj.2021.11.006)
Supplement: Supplementary Material [file mmc1.docx]

**Supplemental material**

Supplemental table 1 Search strategy Ovid EMBASE®

Supplemental table 2 Search strategy Ovid MEDLINE®

Supplemental table 3 Summary of results from studies that have examined device-embedded accelerometry

Supplemental table 4 Summary of results from studies that have examined wearable accelerometry

Supplemental table 5 Validation studies evaluating wearable accelerometry and device-embedded accelerometry

Supplemental table 6 Risk of bias assessment per study according to the six domains of the Quality in Prognostic Studies (QUIPS) tool

Supplemental figure 1 Summary of risk of bias assessment according to the six domains of the Quality in Prognostic Studies (QUIPS) tool

**Supplemental table 1** Search strategy in the Ovid EMBASE®

| **Embase Classic+Embase 2000 to 2020 September 01** | | |  |
| --- | --- | --- | --- |
| # | Searches | Results | Type |
|  | 1 | exp heart failure/ or exp ventricular dysfunction/ or cardiac output, low/ or heart failure with reduced ejection fraction/ or exp heart left ventricle failure/ or exp heart right ventricle failure/ or exp forward heart failure/ or exp congestive heart failure/ | 549906 |
|  | 2 | ((heart or cardiac or myocardial or cardial or ventric* or congestive or systolic or diastolic or output or LV) adj3 (fail* or decompens* or dysfunction* or insufficien* or incompet* or overload* or over-load*)).tw,kw. | 415210 |
|  | 3 | ((low or reduce* or deteriorat* or diminish* or decline* or decrease*) adj2 (EF or LVEF or ejection fraction or left ventricular or output)).tw,kw. | 53337 |
|  | 4 | (hfref or hf-ref or hf or chf).tw,kw. | 107315 |
|  | 5 | exp congestive cardiomyopathy/ or exp tachycardia induced cardiomyopathy/ | 32342 |
|  | 6 | 1 or 2 or 3 or 4 or 5 | 727985 |
|  | 7 | exp heart arrest/ or exp sudden cardiac death/ | 101382 |
|  | 8 | ((sudden or ar?hythm*) adj5 (death or mortality)).tw,kw. | 74470 |
|  | 9 | exp heart ventricle fibrillation/ or exp heart ventricle tachycardia/ or exp heart ventricle flutter/ or exp heart preexcitation/ or exp heart ventricle arrhythmia/ or exp heart muscle conduction disturbance/ or "electrical storm (heart)"/ or polymorphic ventricular tachycardia/ or monomorphic ventricular tachycardia/ or exp heart accessory conduction pathway/ or tachycardia, reciprocating/ or tachycardia, atrioventricular nodal reentry/ or exp paroxysmal tachycardia/ | 269704 |
|  | 10 | (ventric* adj5 (tachyar?hythmia* or tachy-ar?hythmia or fibrillation* or tachycardia* or ar?ythmia or flutter)).mp. | 93313 |
|  | 11 | ((ventricular or ventricle* or life-threat* or LV or shockable) adj3 (fibrillation* or flutter* or tachycardia* or ar?hythmia* or tachyar?hythmia* or tachy-ar?hythmia* or ar?hythmogen* or preexcitat* or pre-excitat*)).tw,kw. | 93150 |
|  | 12 | ((VT or VTs or VF or NSVT) adj4 (episod* or non-sustain* or sustain* or non-monomorphic or monomorphic or polymorphic or RVOT or bidirectional or bi-directional or refractory or recurrent or paroxysmal or pulseless or shockable)).tw,kw. | 8551 |
|  | 13 | electric* storm*.mp. | 1268 |
|  | 14 | exp long QT syndrome/ or exp Brugada syndrome/ or exp heart right ventricle dysplasia/ or exp cardiac channelopathy/ or exp torsade des pointes/ or exp hypertrophic obstructive cardiomyopathy/ or exp hypertrophic cardiomyopathy/ or exp familial hypertrophic cardiomyopathy/ or exp catecholaminergic polymorphic ventricular tachycardia/ or exp progressive cardiac conduction defect/ or exp short qt syndrome/ | 57256 |
|  | 15 | (long adj2 qt adj2 syndrom*).tw,kw. | 8123 |
|  | 16 | (LQT* adj2 syndrome*).tw,kw. | 2971 |
|  | 17 | LQTS.tw,kw. | 2934 |
|  | 18 | torsades de pointes.tw,kw. | 3235 |
|  | 19 | Brugada*.tw,kw. | 5845 |
|  | 20 | (arvc or hocm).tw,kw. | 3735 |
|  | 21 | ((a*yt?m* or hypertrophic or familial or genetic or inherited) adj3 (cardiomyopath* or d?splas*)).tw,kw. | 33434 |
|  | 22 | 7 or 8 or 9 or 10 or 11 or 12 or 13 or 14 or 15 or 16 or 17 or 18 or 19 or 20 or 21 | 432059 |
|  | 24 | ((resynch* or re-synch* or electroversion or cardiover*) adj3 (therap* or device* or defibril*)).tw,kw. | 35488 |
|  | 25 | ((implant* or internal* or automated or intracard* or intra-card* or biventricular or bi-ventricular) adj3 (defibr* or cardioverter* or cardioconver* or resynch* or cardiac device*)).tw,kw. | 30625 |
|  | 26 | (ICDs or AICD or AICDs or CRT-D or CRT or CRTs or CIED or CIEDs).tw,kw. | 44691 |
|  | 27 | (ICD not (ICD-9 or ICD-10 or ICD-11)).tw,kw. | 32081 |
|  | 28 | ((biventricular or bi-ventricular) adj2 (cardioverter or defibril* or pacemaker or pacing or ICD)).tw,kw. | 4141 |
|  | 29 | ((defibril* or cardioverter* or ICD*) adj2 (shock* or therap* or counter-shock*)).tw,kw. | 9979 |
|  | 30 | (anti-tachycardia pac* or antitachycardia pac*).tw,kw. | 1838 |
|  | 31 | 23 or 24 or 25 or 26 or 27 or 28 or 29 or 30 | 146978 |
|  | 32 | exp accelerometer/ or exp activity tracker/ or exp actimetry/ or exp actigraph/ or exp accelerometry/ or exp smart watch/ | 30726 |
|  | 33 | acceleromet*.mp. | 24470 |
|  | 34 | (actigraph* or actograph* or actimeter* or actical or actipal).mp. | 12757 |
|  | 35 | ((mean or raw) adj1 acceleration*).mp. | 375 |
|  | 36 | ((acceleration* or fitne?s or motion or locomotion or inertial or activit* or move* or exercis* or mobility or sleep* or behavio?r* or CSA or PA) adj3 (sensor* or track* or monitor* or device* or detect* or meter*)).tw,kw. | 140052 |
|  | 37 | (Global positioning system* or (GPS adj1 track*)).tw,kw. | 2766 |
|  | 38 | (pedomet* or gyroscop* or magnetomet* or inclinomet*).tw,kw. | 11165 |
|  | 39 | (triaxi* or tri-axi* or single axi* or sing-axi* or dual axi* or dual-axi* or orthogonal axi*).tw,kw. | 5163 |
|  | 40 | (actiwatch or activinsights or geneneactiv or tritrac or GT3X* or GT1M or axivity or garmin or fitbit or smartwatch or apple watch motionwatch or somnitor or somnowatch).tw,kw. | 3623 |
|  | 41 | wearables.mp. | 928 |
|  | 42 | ((wearable or ((wrist or hip or arm or body or leg or thigh) adj1 worn)) adj2 (tracker* or technolog* or device* or monitor* or sensor*)).tw,kw. | 9166 |
|  | 43 | ((activity or step*) adj1 (counter or counting or counts)).tw,kw. | 2833 |
|  | 44 | (d-pa or ((device* or ICD* or CRT) adj3 (PA or activity))).tw,kw. | 1766 |
|  | 45 | ((objective* or device* or longitudinal* or continuous* or external* or direct* or wearable* or algorithm*) adj2 (quantif* or measur* or derive* or asses* or based or analy* or comput* or obtain* or evaluat* or count*) adj3 (activit* or ((sleep or physical) adj2 behavio?r) or movement or PA or sleep* or circadian or cadence or ((ris* or bed or wak*) adj time) or MVPA or sedentary or inactivit* or WASO or energy expenditure or METs or EEact or EEpa or rhythmicity or motion or mobility)).tw,kw. | 14729 |
|  | 46 | 32 or 33 or 34 or 35 or 36 or 37 or 38 or 39 or 40 or 41 or 42 or 43 or 44 or 45 | 199136 |
|  | 47 | 6 or 22 or 31 | 1071643 |
|  | 48 | 46 and 47 | 5526 |
|  | 49 | ((animal/ or animal experiment/ or animal model/ or nonhuman/) not human/) or (cat or cats or porcine or piglet or pig or pigs or rat or rats or dogs or dog or mouse or mice or rodent* or canine).ti. | 7014015 |
|  | 50 | 48 not 49 | 4994 |
|  | 52 | 50 not 51 | 4796 |
|  | 53 | remove duplicates from 52 | 4748 |
|  | 54 | limit 53 to yr="2000 -Current" | 3996 |

**Supplemental table 2** Search strategy in the Ovid MEDLINE®

| **Ovid MEDLINE(R) and Epub Ahead of Print, In-Process & Other Non-Indexed Citations and Daily 2000 to September 01, 2020** | | |  |
| --- | --- | --- | --- |
| Search history sorted by search number ascending | | |  |
|  | # | Searches | Results |
|  | 1 | exp heart failure/ | 122090 |
|  | 2 | exp ventricular dysfunction/ | 37549 |
|  | 3 | Cardiac Output, Low/ | 5509 |
|  | 4 | ((heart or cardiac or myocardium or myocardial or cardial or ventric* or congestive or systolic or diastolic or output or LV) adj3 (fail* or decompens* or dysfunction* or insufficien* or incompet* or overload* or over-load*)).tw,kf. | 245930 |
|  | 5 | ((low or reduce* or deteriorat* or diminish* or decline* or decrease*) adj2 (EF or LVEF or ejection fraction or left ventricular or output)).tw,kf. | 30966 |
|  | 6 | (hfref or hf-ref).tw,kf. | 2068 |
|  | 7 | (hf or chf).tw,kf. | 59462 |
|  | 8 | exp cardiomyopathies/ | 94349 |
|  | 9 | (cardiomyopath* or myocardiopath*).tw,kf. | 74222 |
|  | 10 | exp heart arrest/ | 48193 |
|  | 11 | ((sudden or ar?hythm*) adj5 (death or mortality)).tw,kf. | 49156 |
|  | 12 | ventricular flutter/ | 49 |
|  | 13 | ventricular fibrillation/ | 17020 |
|  | 14 | exp tachycardia, ventricular/ | 16835 |
|  | 15 | (ventric* adj5 (tachyar?hythmia* or tachy-ar?hythmia or fibrillation* or tachycardia* or ar?ythmia or flutter)).mp. | 54299 |
|  | 16 | electric* storm*.mp. | 683 |
|  | 17 | ((ventricular or ventricle* or life-threat* or LV or shockable) adj3 (fibrillation* or flutter* or tachycardia* or ar?hythmia* or tachyar?hythmia* or tachy-ar?hythmia* or ar?hythmogen* or preexcitat* or pre-excitat*)).tw,kf. | 60318 |
|  | 18 | exp Torsades de Pointes/ | 2473 |
|  | 19 | torsades de pointes.tw,kf. | 2347 |
|  | 20 | exp long qt syndrome/ | 7921 |
|  | 21 | (long adj2 qt adj2 syndrom*).tw,kf. | 5476 |
|  | 22 | (LQT* adj2 syndrome*).tw,kf. | 1916 |
|  | 23 | LQTS.tw,kf. | 1787 |
|  | 24 | exp brugada syndrome/ | 3350 |
|  | 25 | Brugada*.tw,kf. | 3717 |
|  | 26 | exp Arrhythmogenic Right Ventricular Dysplasia/ | 2048 |
|  | 27 | (arvc or hocm).tw,kf. | 1871 |
|  | 28 | ((a*yt?m* or hypertrophic or familial or genetic or inherited) adj3 (cardiomyopath* or d?splas*)).tw,kf. | 21548 |
|  | 30 | defibrillators, implantable/ | 16483 |
|  | 31 | cardiac resynchronization therapy/ | 4237 |
|  | 32 | cardiac resynchronization therapy devices/ | 1232 |
|  | 33 | ((resynch* or re-synch* or electroversion or cardiover*) adj3 (therap* or device* or defibril*)).tw,kf. | 21204 |
|  | 34 | ((implant* or internal* or automated or intracard* or intra-card* or biventricular or bi-ventricular) adj3 (defibr* or cardioverter* or cardioconver* or resynch* or cardiac device*)).tw,kf. | 19344 |
|  | 35 | (ICDs or AICD or AICDs or CRT-D or CRT or CRTs or CIED or CIEDs).tw,kf. | 21941 |
|  | 36 | (ICD not (ICD-9 or ICD-10 or ICD-11)).tw,kf. | 15155 |
|  | 37 | ((biventricular or bi-ventricular) adj2 (cardioverter* or defibril* or pacemaker* or pacing or ICD*)).tw,kf. | 2320 |
|  | 38 | ((defibril* or cardioverter* or ICD*) adj2 (shock* or therap* or counter-shock*)).tw,kf. | 5348 |
|  | 39 | (anti-tachycardia pac* or antitachycardia pac*).tw,kf. | 1037 |
|  | 40 | electric countershock/ | 14831 |
|  | 41 | 1 or 2 or 3 or 4 or 5 or 6 or 7 or 8 or 9 | 415786 |
|  | 42 | 10 or 11 or 12 or 13 or 14 or 15 or 16 or 17 or 18 or 19 or 20 or 21 or 22 or 23 or 24 or 25 or 26 or 27 or 28 or 29 | 162621 |
|  | 43 | 30 or 31 or 32 or 33 or 34 or 35 or 36 or 37 or 38 or 39 or 40 | 62115 |
|  | 44 | 41 or 42 or 43 | 564084 |
|  | 45 | exp accelerometry/ | 8948 |
|  | 46 | fitness trackers/ | 574 |
|  | 47 | acceleromet*.mp. | 18198 |
|  | 48 | (actigraph* or actograph* or actimeter* or actical* or actipal*).mp. | 8834 |
|  | 49 | ((mean or raw) adj1 acceleration*).mp. | 293 |
|  | 50 | ((acceleration* or fitne?s or motion or locomotion or inertial or activit* or move* or exercis* or mobility or sleep* or behavio?r* or CSA or PA) adj3 (sensor* or track* or monitor* or device* or detect* or meter*)).tw,kf. | 109972 |
|  | 51 | (Global positioning system* or (GPS adj1 track*)).tw,kf. | 2839 |
|  | 52 | (pedomet* or gyroscop* or magnetomet* or inclinomet*).tw,kf. | 10045 |
|  | 53 | (triaxi* or tri-axi* or single axi* or sing-axi* or dual axi* or dual-axi* or orthogonal axi*).tw,kf. | 4188 |
|  | 54 | (actiwatch or activinsights or geneneactiv or tritrac or GT3X* or GT1M or axivity or garmin or fitbit or smartwatch or apple watch motionwatch or somnitor or somnowatch).tw,kf. | 2300 |
|  | 55 | wearables.mp. | 837 |
|  | 56 | ((wearable or ((wrist or hip or arm or body or leg or thigh) adj1 worn)) adj2 (tracker* or technolog* or device* or monitor* or sensor*)).tw,kf. | 7917 |
|  | 57 | ((activity or step*) adj1 (counter or counting or counts)).tw,kf. | 2016 |
|  | 58 | (d-pa or ((device* or ICD* or CRT) adj3 (PA or activity))).tw,kf. | 1274 |
|  | 59 | ((objective* or device* or longitudinal* or continuous* or external* or direct* or wearable* or algorithm*) adj2 (quantif* or measur* or derive* or asses* or based or analy* or comput* or obtain* or evaluat* or count*) adj3 (activit* or ((sleep or physical) adj2 behavio?r) or movement or PA or sleep* or circadian or cadence or ((ris* or bed or wak*) adj time) or MVPA or sedentary or inactivit* or WASO or energy expenditure or METs or EEact or EEpa or rhythmicity or motion or mobility)).tw,kf. | 10282 |
|  | 60 | 45 or 46 or 47 or 48 or 49 or 50 or 51 or 52 or 53 or 54 or 55 or 56 or 57 or 58 or 59 | 154587 |
|  | 61 | 44 and 60 | 2672 |
|  | 62 | (animals/ not humans/) or (cat or cats or piglet or pig or pigs or porcine or rat or rats or dog or dogs or mouse or mice or rodent* or canine).ti. | 4993461 |
|  | 63 | 61 not 62 | 2319 |
|  | 64 | (expert or current or cochrane or clinical evidence or EBM).jw. or exp guideline/ or exp Databases, Bibliographic/ or editorial/ or books/ or case reports/ or (systematic* adj3 (review or literature)).ti. or ((search* adj12 (literature* or ((electronic or medical or biomedical) adj3 database*) or exhaustiv* or systematic*)) or medline or pubmed or embase or psychinfo or (CENTRAL and cochrane) or "Central Register of Controlled Trials").tw. or (cochrane or clinical evidence or EBM).jw. or ((review/ or meta-analysis/ or (conferenc* or congress*).hw. or (meta analy* or metaanaly* or meta?analy*).ti,ot,kw. or (systematic* adj3 (review or literature)).tw,kw.) not (exp clinical trial/ or comparative study/ or feasibility studies/ or evaluation studies/ or validation studies/ or exp cohort studies/ or cross-sectional studies/ or case-control studies/ or multicenter study/)) | 5483158 |
|  | 65 | 63 not 64 | 1937 |
|  | 66 | remove duplicates from 65 | 1926 |
|  | **67** | **limit 66 to yr="2000 -Current"** | **1437** |

**Supplemental table 3** Summary of results: studies that have examined device-embedded accelerometers

| # | **First author(s) / year** | **Population** | **Vendor** | **Study design** | **Study period** | ***n*** | **Age and gender** | **NYHA** | **LVEF** | **Follow-up^1^** | **Measurement** | **Endpoint** | **Outcome** |
| --- | --- | --- | --- | --- | --- | --- | --- | --- | --- | --- | --- | --- | --- |
| 1 | Auricchio *et al.* 2014 ^1^ | CRT-D | Sorin CRM | Prospective, non-randomised, one-arm trial | 2009- 2011 | 521 | 67.4 ±10.1 y/o, 82% male | II-IV: 99.8% | 25.5 ±7.7% | 17 ±8.7 months | Thresholds adjusted on patient’s baseline after implant | Algorithm to predict HF-related events in the following month | The sensitivity of the prediction algorithm was 34% |
| 2 | Boehmer, *et al.* 2017 ^2^ | CRT-D | Boston Scientific | Data from a non-randomised trial (MultiSENSE) | 2010- 2013 | 974 | 66.8 ±10.3 y/o, 71% male | II-IV: ~95% | 30.0 ±11.4% | 12 months | Thresholds adjusted on patient’s baseline after 45-days of collection | Algorithm to predict HF hospitalisation | The sensitivity of the prediction algorithm was 70% (median lead time before event was 34 days) |
| 3 | Burch, *et al.* 2020 ^3^ | WCD | LifeVest, ZOLL | Retrospective cohort study | 2015- 2018 | 4057 | 60 ±13 y/o, 20% female | Un-known | 23 ±7 | 4 weeks | Step count during WCD use | Shock for sustained VT of VF | Patients who averaged <3637 steps/day during the first week of WCD use were more likely to experience a shock (OR = 4.29, CI 2.58-7.15, P < .001) |
| 4 | Burch, *et al.* 2020 ^4^ | WCD | LifeVest, ZOLL | Retrospective cohort study | 2017 | 4928 | 65 ±13 y/o, 100% female | Un-known | Un-known | 87 [55-113] days | Step count during WCD use | Shock for sustained VT of VF | PA started to decline 16 days before the onset of sustained VT/VF |
| 5 | Chelu *et al.* 2016 ^5^ | ICD or CRT-D | Medtronic | Retrospective observational study | Un-known | 266 | 69 ±10 y/o, 88% male | Un-known | Un-known | 4.3 ±1.5 years | Active minutes per day, the week prior to the first persistent AF episode the following 4 weeks during the AF episode (threshold for activity at 70 steps/min) | Persistent AF onset | Daily PA decreased significantly from the week prior to AF onset compared to each of the 4 consecutive weeks after onset (17% decrease in 16 weeks) |
| 6 | Conraads *et al.* 2014 ^6^ | ICD or CRT-D | Medtronic | Data from prospective studies (SENSE-HF and DOT-HF) | 2005-2009 | 781 | 65 ±10 y/o, 85% men | I (5%), II (49%), III (44%), IV (2%) | 26 ±7 | 15 ±7 months | Average active minutes per day over the earliest 30-day period post-implant (threshold for activity unknown) | Mortality and HF hospitalisation | In multivariable analysis low PA after implantation predicted death and HF hospitalisation |
| 7 | Jame *et al.* (2020) ^7^ | CRT-D (47%) or ICD (53%) | Boston Scientific, Medtronic, Biotronik | Single center retrospective cohort study | 2014- 2016 | 280 | 64.3 y/o, 65% male* | III-IV: 47.5.% | 28.2% | Median 2.4 years | Average active minutes per day standardised to baseline after implant (threshold for activity unknown) | Mortality | Lower baseline PA and a decline in PA were associated with significantly increased risk of mortality |
| 8 | Jame *et al.* (2017) ^8^ | CRT-D | Boston Scientific | Post-hoc analysis of RCT (MADIT-RCT) | 2004- 2008 | 1008 | Age not described, 25% female | Un-known | EF <25%: 63%* | 4.5 years | Average of active minutes per day standardised to a dynamic baseline (threshold for activity at 3.2 km/h). | Mortality | Patients with >40% reduction in PA had a significantly increased 77-day cumulative incidence of death |
| 9 | Jedrzejczyk-Patej *et al.* (2014) ^9^ | CRT-D | Medtronic | Post-hoc analysis of RCT (TRUST CRT) | 2008- 2010 | 96 | ICD-therapy: 62 [58-70] y/o*, 16% female | ICD-therapy: IV: 19%* | ICD-therapy: 23% [20-25]* | 12.03 ±6.7 months | Active minutes per day analysed 10, 7, 3 days and 1 day before ICD intervention (threshold for activity unknown) | Ventricular arrhythmias | Daily PA did not demonstrate significant variances preceding ventricular arrhythmias |
| 10 | Kawabata *et al.* (2007) ^10^ | CRT (83% CRT-D) | Boston Scientific | Prospective observational study | Un-known | 178 | 65 ±8 y/o, 80% male | NYHA III-IV: 100% | 21 ±6 | 668 ±352 days | Active minutes per day, first week after implantation was the baseline (threshold for activity at 3.2 km/h). | None | PA increased after device implantation, a plateau was reached at approximately 12 weeks and thereafter remained stable for up to 2 years |
| 11 | Kelly *et al.* (2020) ^11^ | ICD (32.7%) and CRT-D (67.3%) | Boston Scientific | Registry data (ALTITUDE) | 2007- 2014 | 20927 | 75.7 ±6.2 y/o, 74% male | Unknown | Unknown | 5 years | Average active minutes per day over an 8-week period before event (threshold for activity at 3.2 km/h) | Composite of HF hospitalisation and mortality | A within patient 10-min decrease in average daily PA over an 8-week period from 85 to 75 min was associated with a HR of 4.02 (3.82-4.22) |
| 12 | Kramer *et al.* (2017) ^12^ | CRT-D | Boston Scientific | Registry data (ALTITUDE) | 2008- 2012 | 26509 | 70.2 ±11 y/o, 70.7% male | Unknown | Unknown | 2.3 [1.4-3.4] years | Active minutes per day, 3-10 days after implant as baseline (threshold for activity at 3.2 km/h) | Mortality | Higher 6-month PA change was associated with a lower risk of death (adjusted HR 0.65 per 30 min increase in activity, 95% CI, 0.63–0.67) |
| 13 | Kramer *et al.* (2015) ^13^ | ICD or CRT-D (43.4%) | Boston Scientific | Registry data (ALTITUDE) | 2008- 2012 | 98437 | 67.7 ±13.1 y/o, male 71.7% | Unknown | Unknown | 720 ±437 days | Active minutes per day (threshold for activity at 3.2 km/h) | Mortality | Low baseline (30 to 60 days following implant) and a decline in PA (in any previous 30-day period) were independently associated with a higher risk of death |
| 14 | Lieback *et al.* (2012) ^14^ | ICD | Biotronik | Single center, observational study (Insight-HF) | 2008- 2009 | 32 | 63 ±9 y/o, 84.4% male | III (100%) | 27 ±6 | 164 ±48 days | Continuous measurement following implantation (threshold for activity unknown) | HF hospitalisation | Weight inversely correlated with PA, blood pressure did not correlate with PA. Insufficient number of events to search for potential predictors |
| 15 | Marzec *et al.* (2018) ^15^ | ICD or CRT-D | Boston Scientific | Pilot investigation | Un-known | 235 | Unknown | Unknown | Unknown | 1 year | Active minutes per day (threshold for activity unknown) | VT onset | The predictive accuracy of various machine learning prediction models was no better than random chance (AUC = 0.5) |
| 16 | Palmisano, *et al.* (2018) ^16^ | ICD | Medtronic, Boston Scientific, Biotronik | Data from multicenter registry (IMPLANTED) | 2009- 2016 | 770 | 70.1 ±12.5 y/o, 65.9% male | NYHA II-IV ~ 85% | Low activity: ~34.4 ±11* | 25 [12-50] months | Active minutes per day 30-60 days after implantation (threshold for activity unknown) | Atrial high-rate episodes and composite endpoint of mortality and HF hospitalisation | PA ≥3.5 hours per day was associated with a 38% relative reduction in the risk of atrial high-rate episode and the combined endpoint |
| 17 | Perego et al. (2008) ^17^ | CRT-D | Medtronic | Prospective observational study | 2004- 2007 | 558 | 65 ±11 y/o, 84% male | II: 40%, III: 57%, IV: 3% | 27 ±5 | 326 ±216 days | Active minutes per day, low activity (PA <30 min/day) days counted (threshold for activity unknown) | HF hospitalisation | Patients hospitalised for HF had more low PA days compared to patients who were not hospitalised |
| 18 | Sack *et al*. (2011) ^18^ | CRT-D | Biotronik | Prospective, multicentre observational study (Home-CARE) | 2005- 2008 | 377 | 66.2 ±10 y/o, 78.5% male | 99.2% II-IV | 24.5 ±7.5, ≤35%: 90.7% | 335 ±135 days | Active minutes per day, 25-day monitoring window ending 3 days before event (threshold for activity unknown) | Algorithm to predict cardiovascular hospitalisation and mortality prediction (25-day time window ending 3 days before event) | The sensitivity of the algorithm was 65.4% at 99.5% specificity. The sensitivity for PA alone was 23.6% |
| 19 | Sassone *et al.* (2020) ^19^ | ICD | Boston Scientific | Observational study | Un-known | 24 | 72 ±10 y/o, 71% male | Unknown | Unknown | 80 days | Active minutes per day measured 40 days before and during lockdown (threshold for activity unknown) | None | A 25% mean reduction of PA after lock-down was seen |
| 20 | Sears *et al.* (2018) ^20^ | ICDs or CRT-D | Medtronic | Data from multicenter clinical trial (PainFree SST) | 2009 - 2012 | 2770 | 65 ±12 y/o, 79% male | II-IV: 86% | 32 ±13 | 22 ±9 months | Active minutes per day from implantation onwards (threshold for activity at “walking at slow pace”) | ICD shock or ATP | Daily PA was significantly reduced after an ICD shock, and recovered to normal after 90 days. Pre-shock activity levels did not differ from PA levels of patients who did not receive a shock. |
| 21 | Sears *et al.* (2015) ^21^ | ICD | Medtronic | Post-hoc analysis of RCT (EMPIRIC) | 2002 - 2003 | 174 | 62 ±15 y/o, 82.7% male* | III-IV: 14% | 33 ±14 in shock group* | 1 year | Active minutes per day, 7 days before and 7-days after therapy (threshold for activity at 70 steps/min) | ICD therapy | A significant reduction in activity was observed for few (-26%) and many shocks (-34%) in the first week post-therapy |
| 22 | Sharma *et al.* (2015) ^22^ | CRT-D | Medtronic | Data from prospective observational studies (FAST and PARTNERS-HF) | Un-known | 775 | 69 ±11 y/o, 68% male | II-IV: 99% | EF <35%: 100% | 13 ±5 months | Active minutes per day (threshold for activity at 70 steps/min) | HF hospitalisation within 30 days | The rate of heart failure hospitalisation in the following 30 days was 5.1% if the average PA was at least one week <1hr/day, compared to 1.4% if PA was >1hr/day |
| 23 | Shoemaker *et al.* (2019) ^23^ | ICD or CRT | Medtronic | Retrospective chart study | 2017- 2018 | 168 | 63 ±22.8 y/o, 75% male | I (49%), II (39%) III (12%) | 34.5 ±28.8 | 1 year | Active minutes per day (threshold at 70–80 steps per minute | None | Daily PA seasonal difference between winter and summer months, especially in those with comorbid conditions and an overall activity level of <2.2 hours per day |
| 24 | Shoemaker *et al.* (2012) ^24^ | ICD or CRT-D | Medtronic | Retrospective chart study | 2008- 2011 | 102 | 64.6 ±13.3 y/o, 71% male | 2.0 ±0.76 | 27.8 ± 11.5 | 5 years | Active minutes per day (threshold for activity at 70-80 steps/min) | Mortality | The minimal clinically important difference for a decline in daily PA was approximately 0.5 hours and was 1.0 hours for improvement in daily PA |
| 25 | Singh *et al.* (2009) ^25^ | CRT | Boston Scientific | Data from the CRT RENEWAL and HF-HRV studies | Unknown | 1274 | 66.8+11.8 y/o, 69.5% male | III-IV: 100% | Un-known | Median 11.6 months | Active minutes per day, measured 2 weeks till 3 months following device implant (threshold for activity at 3.2 km/h) | Mortality | A higher PA level 2 weeks post-implantation was associated with a decreased risk of death |
| 26 | Tripp *et al.* (2020) ^26^ | WCD | LifeVest, ZOLL | Retrospective observational study | 2016 | 1952 | 63 [23-90] y/o, 71% male | Un-known | ≤35%: 100% | 90 days | Step count and body position | None | Median step count increased significantly by 67% from the first week of wear to the last week of wear |
| 27 | Vegh *et al.* (2014) ^27^ | CRT | Medtronic and St. Jude Medical | Prospective observational study | 2004- 2010 | 164 | 67.3 ±12.9 y/o, 77% men | II-III: 100% | 25 ±7 | 3 years | Active minutes per day at 1, 3 and 6 months post-implant (Medtronic devices: threshold at 60-70 step per minute, St Jude: Heart rate exceeding resting heart rate) | Composite of HF hospitalisation, LV assist device transplant and all-cause death | Kaplan-Meier curves showed superior freedom from the composite endpoint at 3 year in the highest tertile of PA compared with the lowest tertile |
| 28 | Whellan *et al*. (2010) ^28^ | CRT-D | Medtronic | Prospective, observational study (PARTNER HF) | 2004- 2008 | 694 | 68.4 ±10.7 y/o, 67.3% male | III-IV: 100% | LVEF < 35%: 99.1% | 11.7 ±2.0 months | Active minutes per day (threshold for activity at 70 steps/min) | Algorithm to prediction HF hospitalisation within 1-month | PA was the reason for the algorithm to trigger in 62%, patients with a positive HF algorithm had a 5.5-fold increased risk of HF hospitalisation within the next month |
| 29 | Shakibfar (2019) ^30^ | ICD or CRT-D | Medtronic | Retrospective observational | 2005-2016 | 19935 | Unknown | Unknown | Unknown | 3.5 year | Activity per day (threshold for activity unknown) | Prediction of electrical storms 4 days ahead | Random forest achieved a test accuracy of 0.96 and an area under the curve of 0.80, the percentage of ventricular pacing and the daytime activity were the most relevant variables |
| 30 | Zhao *et al*. (2017) ^29^ | ICD or CRT-D | Biotronik | Retrospective observational (SUMMIT registry) | 2004- 2014 | 845 | Age 60.4 ±14.4 y/o, male 73.7% | III-IV: 49% | 42.6 ±14.9 | 31 ±8.7 months | Active minutes per day measured 30–60 days after implantation (threshold for activity unknown) | Cardiac mortality | There was a significant relationship between low PA after implant and cardiac death (HR 3.644, 95% CI 2.42–5.48, p<0.001) |
| Abbreviations: AF, atrial fibrillation; CI, confidence interval; CRT, cardiac resynchronisation therapy; CRT‐D, CRT defibrillator; HF, heart failure; HFE, heart failure event; HR, hazard ratio; ICD, implantable cardioverter defibrillator; IQR, interquartile range LVEF, left ventricular ejection fraction; MCID, minimal clinically important difference; NYHA, New York Heart Association; PA, physical activity; VTA, ventricular tachyarrhythmia; WCD, wearable cardioverter defibrillator  * Data was described for subgroups only.  ^1^ Follow-up duration provided as mean ±SD or median [IQR] if described in-text | | | | | | | | | | | | | |

**Supplemental table 4** Summary of results: studies that have examined wearable accelerometry

| # | **First author(s) / year** | **Brand accelerometer (site)** | **Population** | **Study design** | **Study period** | **Age and gender** | ***n*** | **Wear time** | **NYHA** | **LVEF** | **Domain** | **Metrics** | **Outcome** |
| --- | --- | --- | --- | --- | --- | --- | --- | --- | --- | --- | --- | --- | --- |
| 31 | Baril *et al* 2019 ^31^ | Fitbit Flex (wrist) | HF | Observational study | 2014-2015 | 55 [47-65], 86% male | 50 | 2 weeks | II-IV: 80% | EF <35%: 100% | Physical activity | Step count | HF patients categorised as NYHA II and III differed significantly in mean of daily total step counts, maximum of daily total steps and mean of daily per minute step count maximums |
| 32 | Cross *et al.* 2010 ^32^ | Actiwatch-64 (wrist) | ICD | Prospective observational study | Unknown | 66.9 ±10.2, 61.5% male | 60 | 14 days | Unknown | ICD: 35.5 ±13.1  CAD:48.7±12.6 | Sleep behaviour | SE, sleep onset latency, WASO and TST | Comparing of ICD patients to a CAD population showed higher mean sleep efficiency in ICD patients compared to CAD. Sleep onset latency, WASO and total sleep time did not differ |
| 33 | Curtis *et al.* 2020^33^ | Actiwatch 2 (wrist) | ICD | Data from randomised controlled trial study design (SAVE) | Unknown | 60.34 ±12.32, 57% male | 37 | 14 days | Unknown | Unknown | Sleep behaviour | SE | SE ratings did not independently predict processing speed task |
| 34 | Curtis *et al.* 2019^34^ | Actiwatch 2 (wrist) | ICD | Data from, randomised controlled trial (SAVE) | Unknown | 60.34 ±12.32, 56% male. | 41 | 14 days | Unknown | Unknown | Sleep behaviour | TST, WASO and SE | TST predicted performance on two tasks of executive function, attentional vigilance and processing speed task. WASO and SE did not predict performance on any test |
| 35 | Da Silva *et al.* 2013 ^35^ | Actigraph GT3X (waist) | HF | Cross sectional | Unknown | 59 ±7.21, 100% male | 16 | 7 days | II-III: 100% | 28.79 ±4.34 | Physical activity | Step count, intensity of PA | Subjects spent 67% of their time in low intensity activities and the average time spent in vigorous intensity activities was very low (4.7%) |
| 36 | Evangelista *et al.* 2010 ^36^ | Sportline Electronic Pedometer, Model 345® (waist) | HF | Data from randomised controlled trial | Unknown | 54 ±12.5, 66.2% male | 71 | 12 months | II-IV: 100% | 26.4 ±6.5 | Physical activity | Distance walked | Participants who increased in daily distance walked during 6-months follow-up had lower risk of the composite endpoint of all-cause hospitalisation and emergency department visits (HR 0.31 95% CI 0.131-0.635), p<0.001) |
| 37 | Gad *et al.*2018 ^37^ | SenseWear Mini Armband (BodyMedia) (wrist) | CRT | Prospective pre-post pilot study | Unknown | 66 ±11, 83% male | 18 | 3.6 ± 0.5 days | Un-known | 23 ±6 pre-CRT | Physical activity | Energy expenditure, steps count, intensity of PA | There was a decrease in total steps per day from pre-CRT to post-CRT |
| 38 | Liebzeit, *et al.* 2017 ^38^ | Actiwatch-64 (wrist) | HF | Comparative cross-sectional | Unknown | HF group 74.40 ±7.49, 60% male, | 40 | 7 days | II-III:100% | 30.85 ±7.9 | Rest-activity patterns | Mesor, amplitude, acrophase and circadian rhythmicity | HF group demonstrated lower 24-hour mean PA (mesor), range of activity during 24h (amplitude), and circadian rhythmicity. Peak of daytime PA (acrophase) was similar between groups |
| 39 | Melin, *et al.* 2016 ^39^ | Actigraph GT3X, (waist) | HF | Prospective observational study | Unknown | Age 70.3, 23.2% female | 60 | 7 days | III: 100% | 23.8% | Physical activity | Sedentary time, intensity of PA, measure of variability | The addition of the peak skewness to the heart-failure survival score (HFSS) model significantly improved the predictive ability |
| 40 | Nguyen *et al.* 2012 ^40^ | Stepwatch 3 (ankle) | HF and ICD | Cross-sectional | 2007- 2010 | 61.1 ±11.6, 86% male | 183 | 7 days | Un-known | EF <35% | Physical activity | Step count, percent time active, intensity of PA, max cadence for 30 continuous, peak performance | ICD patients walked most steps, HF and ICD spent similar time in medium intensity walking. The peak performance was significantly highest in the ICD group, with the highest step rate in 30 minutes |
| 41 | Pozehl *et al.* 2018 ^41^ | Actigraph GT3X, (waist) | HF | Data from randomised trial (HEART Camp trial) | 2016-2018 | 56% male | 182 | 7 days | II-IV: 92.3% | 39.4 ±12.7 | Physical activity | Energy expenditure and intensity of PA | NYHA Class II subjects had significantly more MVPA as compared to Class III, ejection fraction was not related to PA. MVPA had a negative relationship with patient-reported anxiety, whereas MVPA was associated with fewer symptoms, positive attitudes toward exercise, greater exercise self-efficacy and better physical function |
| 42 | Prescher, *et al.* 2016 ^42^ | AiperMotion 300 (waist) | HF | Data from a randomised controlled trial (TIM-HF) | 2008-2010 | 67.7 ±10.8 | 155 | 15 ±6 months | II-III: 100% | Patients with no events: 28.4 ±5.1 | Physical activity | Steps taken during 6MWT, intensity of PA | HF hospitalisation and mortality were more frequent in patients with a lower number of steps and a shorter distance reached during 6MWT at baseline |
| 43 | Redeker, *et al.* 2005 ^43^ | Actiwatch actigraph (wrist) | HF | Cross sectional | Unknown | 59 ±12.85, 39 men, 22 women | 61 | 72 hours | II-IV: 97% | 22.9 ±8% | Sleep behaviour and physical activity | Time in bed, TST, WASO, sleep latency, wake time, duration of wake bouts (nocturnal) activity count | There were no statistically significant correlations between sleep behaviour and mental health, time in bed was negatively associated with functional performance |
| 44 | Redeker, *et al.* 2006^44^ | Actiwatch-64 (wrist) | HF | 2-group comparative design. | Unknown | Age 59.10 ±13.04, 66.1% male | 118 | 3 days | II-IV: 100% | 22.92 ±8.99 | Sleep behaviour | Nocturnal sleep duration, percentage WASO, sleep latency, frequency of wake bouts, duration of wake bouts, and nocturnal activity count | Patients with HF had poorer objective sleep continuity than the comparison group (percentage WASO, frequency of wake bouts, nocturnal activity counts). The duration of sleep was not different between the groups. |
| 45 | Sweeting *et al.* 2018 ^45^ | ActiGraph GT3X+ (waist) | Hypertrophic cardiomyopathy (18% ICD) | Pre- post-trial | 2016-2017 | Age 42 ±13, 60% male | 25 | 7 days | II-IV: 8% | Unknown | Physical activity | Step count, intensity of PA, sedentary time | PA did not increase during a 12-week PA intervention based on control-theory |
| 46 | Sweeting *et al.* 2018 ^46^ | ActiGraph GT3X+ (waist) | Hypertrophic cardiomyopathy (37% ICD) | Cross sectional | 2015 | 48.8 ±14.9, 71% male | 63 | 7 days | II-IV: 100% | Unknown | Physical activity | Step count, intensity of PA | Patients with NYHA II–IV took significantly fewer steps per day compared to NYHA I, no differences were observed across the different intensities of PA |
| 47 | Werhahn, *et al* 2019 ^47^ | Apple Watch (wrist) | HF | Prospective feasibility study | 2017 | Age 46.3 ± 7.8, 60% male | 10 | 2 months | II-IV:100% | 26.7 ±9.8 | Physical activity | Step count | The average steps per day increased over 14 days and correlated significantly with LVEF, 6 min walk test and scores in health-related quality of life |
| 48 | Witham, *et al.* 2006 ^48^ | Stayhealthy RT3 (waist) | HF | Data from RCT | Un-known | 80.5 ±5.0, 55% male | 260 | 21 days (baseline, 3m and 6m) | NYHA II-III: 100% | Unknown | Physical activity | Accelerometer counts | There was no consistent correlation between accelerometry counts and measures of symptoms and NYHA class |
| 49 | Yavari, *et al.*2016 ^49^ | SenseWear Mini Armband (wrist) | HF | Cross sectional | 2010-2014 | Age 72.5 [63-81], 19% female | 151 | 7 days | NYHA I-II: 75% | 37.8 [32.4-49.5] | Physical activity | Energy expenditure, sedentary time, step count, intensity of PA | Sedentary time was higher in the HF groups compared to controls, whereas the control group was most active |
| Abbreviations: CAD: coronary artery disease, CI, confidence interval; CRT, cardiac resynchronisation therapy; CRT‐D, CRT defibrillator; HF, heart failure; HR, hazard ratio; ICD, implantable cardioverter defibrillator; LVEF, left ventricular ejection fraction; NYHA, New York Heart Association; MVPA, moderate-to-vigorous physical activity; PA, physical activity; TST, total sleep time; SE, sleep efficiency; WASO, wake after sleep onset; 6MWT, 6-minute walking test | | | | | | | | | | | | | |

**Supplemental table 5** Summary of results: studies evaluating wearable accelerometry and device-measured accelerometry

| # | **First author(s) / year** | **Device** | **Study design** | **Study period** | **Age and gender** | ***Adherence*** | ***Metrics wearable*** | ***Metrics D-PA*** | ***n*** | **Follow-up** | **NYHA** | **LVEF** | **Outcome** |
| --- | --- | --- | --- | --- | --- | --- | --- | --- | --- | --- | --- | --- | --- |
| 50 | Melczer *et al.* (2016) ^50^ | Biotronik CRT-D or CRT-P (uniaxial) vs. ActiGraph GT3X+ (triaxial) | Registry data | 2015 | 57.35 ±9.54, 18% females | N/A | PA levels (sedentary, lifestyle, moderate, vigorous, very vigorous) | PA average per day (%) | 17 | 7 days | II-III: 100% | 34 ±6.6% | There was a moderate degree of correlation between the values of the device-embedded accelerometer and the wearable accelerometer |
| 51 | Pressler *et al.* (2013) ^51^ | Medtronic ICD or CRT (uniaxial) vs. AiperMotion 440 (triaxial) | Prospective observational study | 2010 | 60 ±20, 79% male | 97% (496 out of 511 days), mean wearing time of 13.7 h/day (range 11–20) | Total daily PA and PA modes ( passive, active (body movement without steps), slow walking (<5 km/h), fast walking (5–7 km/h), and running (>7 km/h)). | Activity minutes per day (threshold approximately 70 steps/min) | 73 | 7 days | II-III: 69% | 31 ±14 | There was a broad variation of total daily activity between both methods resulting in differences in the duration of daily activity up to several hours (95% limits of agreement −225 to 147 min) |
| 52 | Shoemaker *et al.* (2017) ^52^ | Medtronic ICD or CRT-D (uniaxial) vs. ActigraphGT3X (triaxial) | Data from a RCT | 2014-2015 | 64.9 ±11.3, 56.3% male | N/A | Activity counts, step count, and hours or activity per day (overall and based on intensity level of activity) | Activity (hours) per day (threshold activity level equivalent to 70–80 steps per minute). | 16 | 14 days (at baseline and 3-months follow-up) | 2.3 ±0.5 | 31.5 ±10.4% | Approximately 0.80 (95% CI 1.17 to -2.71) hours less activity per day is measured by the device-embedded accelerometer compared with the wearable accelerometer |
| Abbreviations: CI, confidence interval; CRT, cardiac resynchronisation therapy; CRT‐D, CRT defibrillator; ICD, implantable cardioverter defibrillator | | | | | | | | | | | | | |

**Supplemental table 6** Risk of bias assessment per study according to the six domains of the Quality in Prognostic Studies (QUIPS) tool

|  | **1. Study Participation** | **2. Study Attrition** | **3. Prognostic Factor Measurement** | **4. Outcome Measurement** | **5. Study Confounding** | **6. Statistical Analysis and Reporting** | **Overall risk of bias** |
| --- | --- | --- | --- | --- | --- | --- | --- |
| Auricchio *et al.* 2014 ^1^ |  |  |  |  |  |  |  |
| Baril *et al.* 2019 ^31^ |  |  |  |  |  |  |  |
| Boehmer *et al.* 2017 ^2^ |  |  |  |  |  |  |  |
| Burch *et al.*2020 ^4^ |  |  |  |  |  |  |  |
| Burch *et al.* 2019 et al. ^3^ |  |  |  |  |  |  |  |
| Chelu *et al.* 2016 ^5^ |  |  |  |  |  |  |  |
| Conraads *et al.* 2014 ^6^ |  |  |  |  |  |  |  |
| Cross *et al.* 2010 ^32^ |  |  |  |  |  |  |  |
| Curtis *et al.* 2020 ^33^ |  |  |  |  |  |  |  |
| Curtis *et al.* 2019 ^34^ |  |  |  |  |  |  |  |
| Da Silva *et al.* 2013 ^35^ |  |  |  |  |  |  |  |
| Evangalista *et al.* 2010 ^36^ |  |  |  |  |  |  |  |
| Gad *et al.* 2018 ^37^ |  |  |  |  |  |  |  |
| Jame *et al.* 2017 ^8^ |  |  |  |  |  |  |  |
| Jame *et al.* 2020 ^53^ |  |  |  |  |  |  |  |
| Jedrzejczyk-Patej *et al.* 2014 ^54^ |  |  |  |  |  |  |  |
| Kawabata *et al.* 2007 ^55^ |  |  |  |  |  |  |  |
| Kelly *et al.* 2020 ^11^ |  |  |  |  |  |  |  |
| Kramer *et al.* 2015 ^13^ |  |  |  |  |  |  |  |
| Kramer *et al.* 2017 ^12^ |  |  |  |  |  |  |  |
| Lieback *et al.* 2012 ^14^ |  |  |  |  |  |  |  |
| Liebzeit *et al.* 2017 ^38^ |  |  |  |  |  |  |  |
| Marzec *et al.* 2018 ^15^ |  |  |  |  |  |  |  |
| Melin *et al.* 2016 ^39^ |  |  |  |  |  |  |  |
| Ngyuen *et al.* 2012 ^40^ |  |  |  |  |  |  |  |
| Palmisano *et al.* 2018^16^ |  |  |  |  |  |  |  |
| Perego *et al.* 2008 ^56^ |  |  |  |  |  |  |  |
| Pozehl *et al.* 2018 ^41^ |  |  |  |  |  |  |  |
| Prescher *et al.* 2016 ^42^ |  |  |  |  |  |  |  |
| Redeker *et al.* 2005 ^43^ |  |  |  |  |  |  |  |
| Redeker *et al.* 2006 ^44^ |  |  |  |  |  |  |  |
| Sack *et al.* 2011 ^18^ |  |  |  |  |  |  |  |
| Sassone *et al.* 2020 ^19^ |  |  |  |  |  |  |  |
| Shoemaker *et al.* ^23^ |  |  |  |  |  |  |  |
| Shoemaker *et al.* 2012 ^24^ |  |  |  |  |  |  |  |
| Sears *et al.* 2015 ^21^ |  |  |  |  |  |  |  |
| Sears *et al.* 2018 ^20^ |  |  |  |  |  |  |  |
| Shakibfar *et al.* (2019) ^30^ |  |  |  |  |  |  |  |
| Sharma *et al.* 2015 ^22^ |  |  |  |  |  |  |  |
| Singh *et al.* 2009 ^25^ |  |  |  |  |  |  |  |
| Sweeting *et al.* 2018 ^45^ |  |  |  |  |  |  |  |
| Sweeting *et al.* 2018 ^46^ |  |  |  |  |  |  |  |
| Tripp *et al.* 2020 ^26^ |  |  |  |  |  |  |  |
| Vegh *et al.* 2014 ^27^ |  |  |  |  |  |  |  |
| Werhahn *et al.* 2019 ^47^ |  |  |  |  |  |  |  |
| Whellan *et al.* 2010 ^28^ |  |  |  |  |  |  |  |
| Witham *et al.* 2006 ^48^ |  |  |  |  |  |  |  |
| Yavari et al. 2017 ^49^ |  |  |  |  |  |  |  |
| Zhao *et al.* 2017 ^29^ |  |  |  |  |  |  |  |

= Low risk of bias

= Moderate risk of bias

= Unknown

= High risk of bias

**Supplemental figure 1** Summary of risk of bias assessment according to the six domains of the Quality in Prognostic Studies (QUIPS) tool


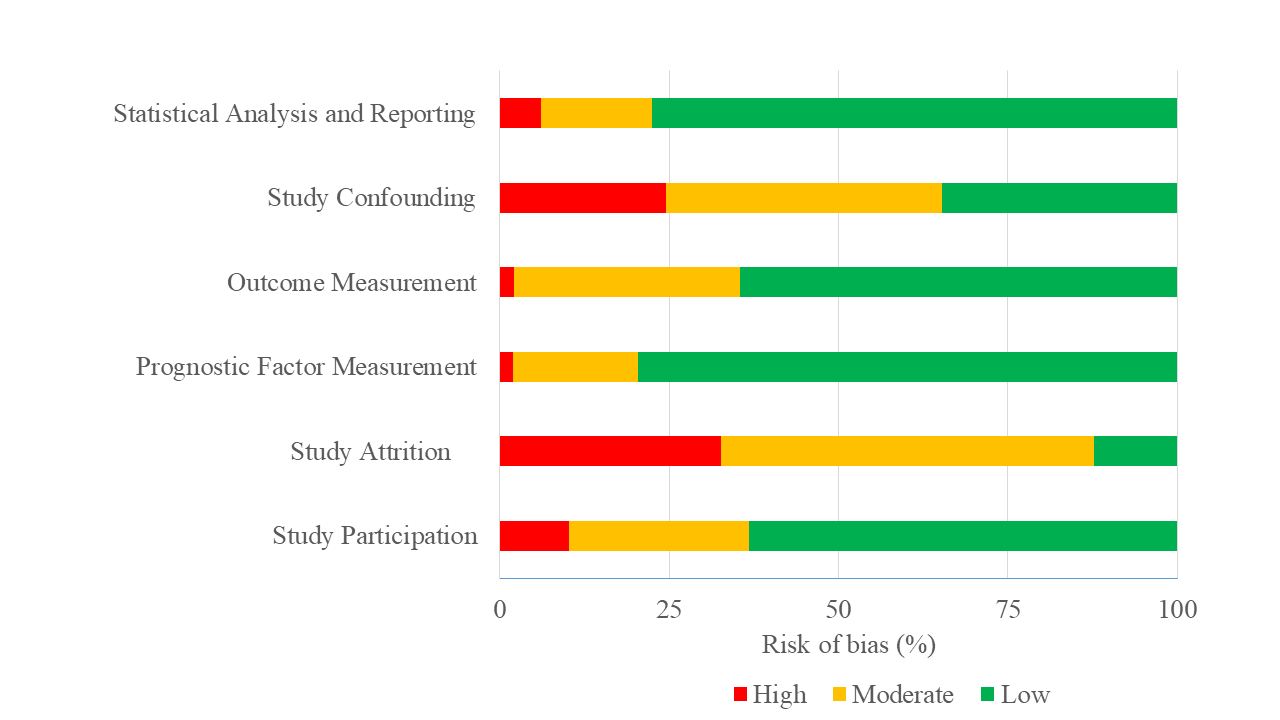


**References**

[1] Auricchio A, Gold MR, Brugada J, Nolker G, Arunasalam S, Leclercq C, et al. Long-term effectiveness of the combined minute ventilation and patient activity sensors as predictor of heart failure events in patients treated with cardiac resynchronization therapy: Results of the Clinical Evaluation of the Physiological Diagnosis Function in the PARADYM CRT device Trial (CLEPSYDRA) study. *Eur J Heart Fail* 2014; **16**: 663-670.

[2] Boehmer JP, Hariharan R, Devecchi FG, Smith AL, Molon G, Capucci A, et al. A Multisensor Algorithm Predicts Heart Failure Events in Patients With Implanted Devices: Results From the MultiSENSE Study. *JACC Heart Fail* 2017; **5**: 216-225.

[3] Burch AE, D'Souza B, Gimbel JR, Rohrer U, Masuda T, Sears S, et al. Physical activity is reduced prior to ventricular arrhythmias in patients with a wearable cardioverter defibrillator. *Clin Cardiol* 2020; **43**: 60-65.

[4] Burch AE, Erath JW, Kutyifa V, Assmus B, Bonderman D, Russo AM. Decline in physical activity in the weeks preceding sustained ventricular arrhythmia in women. *Heart Rhythm O2* 2020; **1**: 283-287.

[5] Chelu MG, Gunderson BD, Koehler J, Ziegler PD, Sears SF. Patient Activity Decreases and Mortality Increases After the Onset of Persistent Atrial Fibrillation in Patients With Implantable Cardioverter-Defibrillators. *JACC Clin Electrophysiol* 2016; **2**: 518-523.

[6] Conraads VM, Spruit MA, Braunschweig F, Cowie MR, Tavazzi L, Borggrefe M, et al. Physical activity measured with implanted devices predicts patient outcome in chronic heart failure. *Circ Heart Fail* 2014; **7**: 279-287.

[7] Jame S, Cascino T, Yeow R, Ananwattanasuk T, Ghannam M, Coatney J, et al. Baseline and decline in device-derived activity level predict risk of death and heart failure in patients with an ICD for primary prevention. *Pacing Clin Electrophysiol* 2020.

[8] Jame S, Kutyifa V, Polonsky B, McNitt S, Al-Ahmad A, Moss AJ, et al. Predictive value of device-derived activity level for short-term outcomes in MADIT-CRT. *Heart Rhythm* 2017; **14**: 1081-1086.

[9] Jedrzejczyk-Patej E, Kowalski O, Sredniawa B, Pruszkowska P, Sokal A, Szulik M, et al. Trying to predict the unpredictable: Variations in device-based daily monitored diagnostic parameters can predict malignant arrhythmic events in patients undergoing cardiac resynchronization therapy. *Cardiol J* 2014; **21**: 405-412.

[10] Kawabata MF, C.; Regoli, F.; Raffa, S.; Pastori, F.; Fratini, S.; Prentice, J.; Klein, H.U.; Auricchio, A. Activity Monitoring in Heart Failure Patients With Cardiac Resynchronization Therapy2007.

[11] Kelly JP, Ballew NG, Lin L, Hammill BG, Stivland TM, Jones PW, et al. Association of Implantable Device Measured Physical Activity With Hospitalization for Heart Failure. *JACC Heart Fail* 2020; **8**: 280-288.

[12] Kramer DB, Jones PW, Rogers T, Mitchell SL, Reynolds MR. Patterns of physical activity and survival following cardiac resynchronization therapy implantation: the ALTITUDE activity study. *Europace* 2017; **19**: 1841-1847.

[13] Kramer DB, Mitchell SL, Monteiro J, Jones PW, Normand SL, Hayes DL, et al. Patient Activity and Survival Following Implantable Cardioverter-Defibrillator Implantation: The ALTITUDE Activity Study. *J Am Heart Assoc* 2015; **4**.

[14] Lieback A, Proff J, Wessel K, Fleck E, Gotze S. Remote monitoring of heart failure patients using implantable cardiac pacing devices and external sensors: results of the Insight-HF study. *Clin Res Cardiol* 2012; **101**: 101-107.

[15] Marzec L, Raghavan S, Banaei-Kashani F, Creasy S, Melanson EL, Lange L, et al. Device-measured physical activity data for classification of patients with ventricular arrhythmia events: A pilot investigation. *PLoS One* 2018; **13**: e0206153.

[16] Palmisano P, Guerra F, Ammendola E, Ziacchi M, Luigi Pisano EC, Dell'Era G, et al. Physical Activity Measured by Implanted Devices Predicts Atrial Arrhythmias and Patient Outcome: Results of IMPLANTED (Italian Multicentre Observational Registry on Patients With Implantable Devices Remotely Monitored). *J Am Heart Assoc* 2018; **7**.

[17] Perego GB, Landolina M, Vergara G, Lunati M, Zanotto G, Pappone A, et al. Implantable CRT device diagnostics identify patients with increased risk for heart failure hospitalization. *Journal of Interventional Cardiac Electrophysiology* 2008; **23**: 235-242.

[18] Sack S, Wende CM, Nagele H, Katz A, Bauer WR, Barr CS, et al. Potential value of automated daily screening of cardiac resynchronization therapy defibrillator diagnostics for prediction of major cardiovascular events: results from Home-CARE (Home Monitoring in Cardiac Resynchronization Therapy) study. *Eur J Heart Fail* 2011; **13**: 1019-1027.

[19] Sassone B, Mandini S, Grazzi G, Mazzoni G, Myers J, Pasanisi G. Impact of COVID-19 Pandemic on Physical Activity in Patients With Implantable Cardioverter-Defibrillators. *J Cardiopulm Rehabil Prev* 2020; **40**: 285-286.

[20] Sears SF, Rosman L, Sasaki S, Kondo Y, Sterns LD, Schloss EJ, et al. Defibrillator shocks and their effect on objective and subjective patient outcomes: Results of the PainFree SST clinical trial. *Heart Rhythm* 2018; **15**: 734-740.

[21] Sears SF, Whited A, Koehler J, Gunderson B. Examination of the differential impacts of antitachycardia pacing vs. shock on patient activity in the EMPIRIC study. *Europace* 2015; **17**: 417-423.

[22] Sharma V, Rathman LD, Small RS, Whellan DJ, Koehler J, Warman E, et al. Stratifying patients at the risk of heart failure hospitalization using existing device diagnostic thresholds. *Heart Lung* 2015; **44**: 129-136.

[23] Shoemaker MJ, Ferrick A, Fischer C, Schuurman C, Cartwright K, McLeod J, et al. Quantification of Seasonal Variation in Daily Physical Activity in Individuals with Heart Failure and Implantable Cardioverter Defibrillator/Cardiac Resynchronisation Therapy Devices. *Heart International* 2019; **13**.

[24] Shoemaker MJ, Curtis AB, Vangsnes E, Dickinson MG, Paul R. Analysis of daily activity data from implanted cardiac defibrillators: The minimum clinically important difference and relationship to mortality/life expectancy. *World Journal of Cardiovascular Diseases* 2012; **02**: 129-135.

[25] Singh JP, Rosenthal LS, Hranitzky PM, Berg KC, Mullin CM, Thackeray L, et al. Device diagnostics and long-term clinical outcome in patients receiving cardiac resynchronization therapy. *Europace* 2009; **11**: 1647-1653.

[26] Tripp C, Burch AE, Erath JW, Hain A, Sears SF. Physical Activity in Adults With Wearable Cardioverter Defibrillators in the Post-Myocardial Infarction Period. *J Cardiopulm Rehabil Prev* 2020; **40**: 164-166.

[27] Vegh EM, Kandala J, Orencole M, Upadhyay GA, Sharma A, Miller A, et al. Device-measured physical activity versus six-minute walk test as a predictor of reverse remodeling and outcome after cardiac resynchronization therapy for heart failure. *Am J Cardiol* 2014; **113**: 1523-1528.

[28] Whellan DJ, Ousdigian KT, Al-Khatib SM, Pu W, Sarkar S, Porter CB, et al. Combined heart failure device diagnostics identify patients at higher risk of subsequent heart failure hospitalizations: results from PARTNERS HF (Program to Access and Review Trending Information and Evaluate Correlation to Symptoms in Patients With Heart Failure) study. *J Am Coll Cardiol* 2010; **55**: 1803-1810.

[29] Zhao S, Chen K, Su Y, Hua W, Chen S, Liang Z, et al. Association between patient activity and long-term cardiac death in patients with implantable cardioverter-defibrillators and cardiac resynchronization therapy defibrillators. *Eur J Prev Cardiol* 2017; **24**: 760-767.

[30] Shakibfar S, Krause O, Lund-Andersen C, Aranda A, Moll J, Andersen TO, et al. Predicting electrical storms by remote monitoring of implantable cardioverter-defibrillator patients using machine learning. *Europace* 2019; **21**: 268-274.

[31] Baril JF, Bromberg S, Moayedi Y, Taati B, Manlhiot C, Ross HJ, et al. Use of Free-Living Step Count Monitoring for Heart Failure Functional Classification: Validation Study. *JMIR Cardio* 2019; **3**: e12122.

[32] Cross NJ, McCrae CS, Smith KM, Conti JB, Sears SF. Comparison of actigraphic and subjective measures of sleep in implantable cardioverter defibrillator and coronary artery disease patients. *Clin Cardiol* 2010; **33**: 753-759.

[33] Curtis AF, Roth AJ, Sears SF, Conti JB, Berry RB, Dzierzewski JM, et al. Associations between pain, objective sleep efficiency and cognition in patients with implantable cardioverter defibrillators. *Sleep Med* 2020; **72**: 122-125.

[34] Curtis AF, Roth AJ, Sears SF, Conti JB, Berry RB, Dzierzewski JM, et al. Cognitive performance in patients with implantable cardioverter defibrillators: Associations with objective sleep duration, age and anxiety. *J Sleep Res* 2019; **28**: e12810.

[35] da Silva VZ, Lima AC, Vargas FT, Cahalin LP, Arena R, Cipriano G, Jr. Association between physical activity measurements and key parameters of cardiopulmonary exercise testing in patients with heart failure. *J Card Fail* 2013; **19**: 635-640.

[36] Evangelista LS, Hamilton MA, Fonarow GC, Dracup K. Is exercise adherence associated with clinical outcomes in patients with advanced heart failure? *Phys Sportsmed* 2010; **38**: 28-36.

[37] Gad SA, Martin S, Kimber S, Williams R, Gulamhusein S, Lockwood E, et al. Impact of Cardiac Resynchronization Therapy on Daily Physical Activity in Heart Failure Patients. *J Cardiopulm Rehabil Prev* 2018; **38**: E1-E4.

[38] Liebzeit D, Phelan C, Moon C, Brown R, Bratzke L. Rest-Activity Patterns in Older Adults with Heart Failure and Healthy Older Adults. *J Aging Phys Act* 2017; **25**: 116-122.

[39] Melin M, Hagerman I, Gonon A, Gustafsson T, Rullman E. Variability in Physical Activity Assessed with Accelerometer Is an Independent Predictor of Mortality in CHF Patients. *PLoS One* 2016; **11**: e0153036.

[40] Nguyen HQ, Steele BG, Dougherty CM, Burr RL. Physical activity patterns of patients with cardiopulmonary illnesses. *Arch Phys Med Rehabil* 2012; **93**: 2360-2366.

[41] Pozehl BJ, McGuire R, Duncan K, Hertzog M, Deka P, Norman J, et al. Accelerometer-Measured Daily Activity Levels and Related Factors in Patients With Heart Failure. *J Cardiovasc Nurs* 2018; **33**: 329-335.

[42] Prescher S, Schoebel C, Koehler K, Deckwart O, Wellge B, Honold M, et al. Prognostic value of serial six-minute walk tests using tele-accelerometry in patients with chronic heart failure: A pre-specified sub-study of the TIM-HF-Trial. *Eur J Prev Cardiol* 2016; **23**: 21-26.

[43] Redeker NS, Hilkert R. Sleep and quality of life in stable heart failure. *J Card Fail* 2005; **11**: 700-704.

[44] Redeker NS, Stein S. Characteristics of sleep in patients with stable heart failure versus a comparison group. *Heart Lung* 2006; **35**: 252-261.

[45] Sweeting J, Ingles J, Ball K, Semsarian C. A Control Theory-Based Pilot Intervention toIncrease Physical Activity in Patients WithHypertrophic Cardiomyopathy. *Am J Cardiol* 2018; **122**: 866-871.

[46] Sweeting J, Ingles J, Ball K, Semsarian C. Daily Step Count as a Simple Marker of Disease Severity in Hypertrophic Cardiomyopathy. *Heart Lung Circ* 2018; **27**: 752-755.

[47] Werhahn SM, Dathe H, Rottmann T, Franke T, Vahdat D, Hasenfuss G, et al. Designing meaningful outcome parameters using mobile technology: a new mobile application for telemonitoring of patients with heart failure. *ESC Heart Fail* 2019; **6**: 516-525.

[48] Witham MD, Argo IS, Johnston DW, Struthers AD, McMurdo ME. Predictors of exercise capacity and everyday activity in older heart failure patients. *Eur J Heart Fail* 2006; **8**: 203-207.

[49] Yavari M, Haykowsky MJF, Savu A, Kaul P, Dyck JRB, Haennel RG, et al. Volume and Patterns of Physical Activity Across the Health and Heart Failure Continuum. *Can J Cardiol* 2017; **33**: 1465-1471.

[50] Melczer C, Melczer L, Goják I, Oláh A, Ács P. A comparative analysis between external accelerometer and internal accelerometer’s physical activity data from implanted resynchronization devices in patients with heart failure. *European Journal of Integrative Medicine* 2016; **8**: 18-22.

[51] Pressler A, Danner M, Esefeld K, Haller B, Scherr J, Schomig A, et al. Validity of cardiac implantable electronic devices in assessing daily physical activity. *Int J Cardiol* 2013; **168**: 1127-1130.

[52] Shoemaker MJ, Cartwright K, Hanson K, Serba D, Dickinson MG, Kowalk A. Concurrent Validity of Daily Activity Data From Medtronic ICD/CRT Devices and the Actigraph GT3X Triaxial Accelerometer. *Cardiopulmonary Physical Therapy Journal* 2017; **28**: 3-11.

[53] Jame S, Cascino T, Yeow R, Ananwattanasuk T, Ghannam M, Coatney J, et al. Baseline and decline in device-derived activity level predict risk of death and heart failure in patients with an ICD for primary prevention. *Pacing Clin Electrophysiol* 2020; **43**: 775-780.

[54] Jędrzejczyk-Patej E, Kowalski O, Średniawa B, Pruszkowska P, Sokal A, Szulik M, et al. Trying to predict the unpredictable: Variations in device-based daily monitored diagnostic parameters can predict malignant arrhythmic events in patients undergoing cardiac resynchronization therapy. *Cardiology Journal* 2014; **21**: 405-412.

[55] Kawabata M, Fantoni C, Regoli F, Raffa S, Pastori F, Fratini S, et al. Activity Monitoring in Heart Failure Patients With Cardiac Resynchronization Therapy. *Circ J* 2007; **71**: 1885 - 1892.

[56] Perego GB, Landolina M, Vergara G, Lunati M, Zanotto G, Pappone A, et al. Implantable CRT device diagnostics identify patients with increased risk for heart failure hospitalization. *J Interv Card Electrophysiol* 2008; **23**: 235-242.
